# Images of synthetic life: Mapping the use and function of metaphors in the public discourse on synthetic biology

**DOI:** 10.1371/journal.pone.0199597

**Published:** 2018-06-21

**Authors:** Matthias Braun, Sandra Fernau, Peter Dabrock

**Affiliations:** Chair of Systematic Theology II (Ethics), Friedrich-Alexander-Universität Erlangen-Nürnberg, Erlangen, Germany; Indiana University Bloomington, UNITED STATES

## Abstract

Synthetic biology is currently one of the most frequently addressed emerging biotechnologies. Developments within this field receive a great deal of attention in media coverage, in which they are frequently illustrated by certain forms of metaphorical speech. Although it can be assumed that societal perceptions and evaluations of emerging biotechnologies are shaped by media coverage and its transported images, there is a lack of empirical research examining the reporting on synthetic biology as well as the use and function of metaphors within media articles. Thus, filling in this gap is one of the urgent desiderata for gaining an enhanced understanding of public views and assessments of this field of biotechnology. Against this background, this article addresses two main questions: (1) Which metaphors and framings are prevalent in the media discourse and what meaning do they have? (2) In which way are metaphors used in media coverage and what function do they have? The research is based on a media content analysis and includes a total number of 11.867 German- as well as English-language media articles dealing with synthetic biology, covering the period between 2004 and 2015. The findings suggest that forms of metaphorical speech address the novelty of current and envisioned scientific developments, highlighting their potential to shift social values and cultural concepts of life and nature. Basic expressions for describing progress within the field of synthetic biology are mainly descriptive metaphors originating from the semantic fields of craft, engineering, IT or art. In comparison, the total frequency of religiously charged metaphors, such as “playing God” or “creating life”, is substantially lower. This low usage rate of religio-cultural expressions in media coverage can be considered a surprising result, since other empirical studies and particularly the ongoing broader ethical discussion attach more importance to these forms of metaphorical speech.

## Introduction

Emerging biotechnologies operate at the interface between science and society, evoking both manifold expectations as well as concerns from a broader public. Synthetic biology (SB) is one of the most frequently and controversially debated biotechnologies, receiving a great deal of attention from scientists, policy makers, civil society organissations as well as in media coverage. Metaphors and figures of speech are important parts of the public discourse in general, and the media discourse on science and technology in particular. As such, they perform functions of explaining and persuading, often being used to describe general trends, but also for giving reasons for adopted positions or assumed courses of action regarding several scientific developments. By providing alleged justifications for subjective perceptions, metaphors may serve as markers of consensus or controversy between social actors with regard to the handling of developments within the field of SB [1,2,3,4]. This raises questions about which metaphors occur in media discourse to promote particular viewpoints as well as about the manner and contexts of their usage.

Synthetic biology is widely understood as an umbrella term covering a diverse field of scientific practices with different agendas and approaches. Varied disciplines are combined within its framework, such as physical, chemical and computer sciences, engineering and biology. The general objective is either to construct and design new biological parts, devices and systems (the bottom-up approach), or to modulate and modify existing natural components by implementing, for example, synthetic genes or proteins (the top-down approach) [5].

The underlying conceptual approach of SB is to gain a more in-depth and accurate understanding of biological systems. Therefore, SB addresses a well-defined understanding of the organisational principles of biological organisms. By using a methodological framework of prediction, analysis, modulation, as well as by building new biological components SB tries to conceptualise and finally create new modularised biological systems. Thus, SB is perhaps more precisely understood if it is seen as a platform of different interacting bio-technological tools and newly modularised and constructed reagents. In the long term, the different approaches of SB aim to develop novel and wide-ranging applications within a variety of societal fields, offering new applications in health care, agriculture, food production, and environmental protection [6,7]. Beyond the first achievements within a synthetic version of the antimalarial compound artemisinin there are several projects within SB, which sustainably aim at contributing to the fight against different communicable diseases such as the Human Immunodeficiency Virus or to enhance the Hepatitis C Virus vaccine development.

Beside the envisioned benefits of SB, its emerging advances are supposed to evoke new challenges for science and society. In the current public discussion, a special focus is put on the visions and (postulated) products of SB, emphasising the objective of generating new forms of life. These future aims, as well as the related successes proclaimed by scientists within this field, show a potential rupture of cultural concepts of life and nature, blurring the classical distinctions between ‘living’ and ‘non-living’ as well as ‘natural’ and ‘artificial’ [8,9]. Such possible developments challenge societal norms and values and may have substantial implications for shared ways of life. Against this background, an important task is to examine the prevalence, manner of use, and function of images of synthetic in public discourse in order to attain a better understanding of modes of societal perception of emerging biotechnologies.

While a large number of studies have explored public views on different biotechnologies, those that focus on SB are still few in number. The research conducted on this subject consists primarily of surveys, focus group and interview studies with selected or representatively polled populations of different countries from the EU [10–12] or the US [13–16]. In spite of the widespread recognition that the societal assessment of emerging biotechnologies is heavily shaped by media coverage and its transported images [2,4,17,18], there is a lack of empirical research examining the reporting on SB as well as the use and function of metaphors within media articles.

On a general level, studies of metaphors in public discourse on biotechnologies reveal two key aspects: On the one hand, the findings indicate that forms of metaphorical speech have been frequently used in international media coverage and have therefore shaped media representations of these scientific fields [1,2,14,19]. On the other hand, the results of studies on the media coverage of biotechnology in different countries show that the use of metaphors likely varies from one language to the next. A media content analysis on the use of metaphor in Danish media coverage of biotechnology from 2005 to 2006 points out that such expressions are grounded in basic schematic images and fulfil important tasks for rendering a common level of comprehension. At the same time, they are highly influenced by notions of risk and anxiety. According to this analysis, emotionally charged metaphors, referring to religious semantics and especially to Frankenstein scenarios, figure quite often in the examined Danish media reports [1]. Hence, these findings indicate that, in media discourse in Denmark, (emerging) biotechnologies are represented in an over-simplified and sceptical manner. This is consistent with the quite frequently occurring general presumption of rather reductionist and emotionally appealing reporting on science and technology [2,14]. By contrast, a content analysis of German-language media articles with a focus on SB, published between 2004 and 2008, indicates that this research field was broadly represented with regard to its envisioned benefits, and thus, in a simplifying but rather affirmative manner [19]. Nevertheless, this study also shows that religio-culturally charged metaphors, such as “playing God” or “creation”, can be frequently found in media reports. However, phrases with mechanical and industrial references are most prevalent in public discourse on SB in the observed period [19,20]. The empirically observed high usage of metaphorical expressions correspond to the ongoing broad theoretical discussion on metaphors in the field of SB [8,9,21–23], which focuses on the occurrence of religiously charged and emotionally appealing expressions, and aims at analysing and assessing SB and its notions of life from a philosophical, ethical or theological perspective.

Nevertheless, although the use of metaphors in public discourse on SB can be considered a widely debated issue on a theoretical level, the frequency of their occurrence, as well as the manner in which they are used and their function in media coverage, is currently insufficiently explored, especially with regard to international media reporting on SB. Therefore, filling in this gap is one of the urgent desiderata for gaining an enhanced understanding of public views and evaluations of this highly debated and controversial field of research. Against this background, the present study explores the societal awareness of SB by systematically analysing and interpreting the usage and function of metaphors that are prevalent in German- as well as English-language media coverage on SB. Thus, the main questions to be addressed by this article are the following: (1) which metaphors are prevalent in the media discourse on SB and what meaning do they have? (2) How are the metaphors used in media coverage on SB and what function do they have?

## Methods

The underlying assumption of our investigation is that leading metaphors in media reporting contribute to shaping public perceptions and assessments of emerging biotechnologies by framing and structuring related discourses and creating powerful images of actors and (possible) products within these fields [2,17,24]. Following the Lakoff & Johnson [25] approach to the theory of metaphor, metaphors are not only figures of speech but also forms which express the exterior of those metaphorical concepts, guiding the interpretation and organisation of reality. As “speech acts” [26], metaphors additionally have structuring functions, providing orientation and leading to action [25]. Although it is important to note that public views cannot be predicted based on media content analysis alone, the analysis of metaphors in public discourse is an appropriate approach for scrutinising public communication patterns as well as societal modes of perception of emerging biotechnologies. Furthermore, by scrutinizing the use of metaphors and speech forms it is possible to gain a better understanding of the implicitly underlying cultural perceptions which are addressed and perhaps be challenged by a new technology such as SB.

With this in mind, we conducted a media content analysis [27] that explores the use and function of imaginations and metaphors in the discourse on SB. The analysis included national (German-language) as well as international (English-language) media articles dealing with SB, covering the period from January 2004 to December 2015. This sample period was fixed at the first international conference on SB, which took place in 2004, and ends up with the current development of SB as a more or less established field of research.

In a first step, regarding the use of metaphorical forms of speech in German-language media coverage of SB, we searched “GBI-Genios”, the market-leading media database in Germany, which is comprised of 340 daily and weekly magazines (e.g. Frankfurter Allgemeine Zeitung, Süddeutsche Zeitung, Das Handelsblatt, Der Spiegel, Die Zeit). The selected search term was “Synthetische Biologie”. In order to examine the use of metaphor in international media coverage on SB, in a second step the database “LexisNexis” was searched, again using the keyword “Synthetic Biology”. As one of the largest international research databases, “LexisNexis” covers a broad spectrum of more than 1.000 daily and weekly, local, national, and international publications (e.g. The New York Times, The Washington Post, The Financial Times, The Economist, The Guardian). The overall sample included articles published in many different countries worldwide as well as different print and online media genres (e.g. newspapers, journals, press reports or popular science newsletters). It consisted of a varied and comprehensive set of press material with regard to expected target groups, patterns of interpretation or argumentation and transported values, thus providing a basis for a generalizable analysis of metaphor use in public discourse.

We decide to use a combination of quantitative [28,29] and qualitative [30,31] content analysis to get a large-scaled and in-depth view of how the field of SB was covered by the media. All media articles were analysed first by each investigator and in a second step within the research team in order to reach a broad intercoder consensus. The aim of the quantitative content analysis was to examine which metaphors and framings—as a hint to the implicit used and referred to comprehensive cultural perceptions—are prevalent in the public discourse and guide perceptions and evaluations of SB. In this context, the temporal frequency distribution (rate of increase respectively decrease) was also investigated. Within the scope of the prior quantitative content analysis of German-language media coverage, the most-used metaphors were inductively extracted based on specific findings from the articles to ensure representing a large spectrum of metaphors dealt with in media coverage. The thereby operationalized list of metaphors which were generally used in the media discourse on SB was then used as a basis both for the inquiry into the international media coverage and the qualitative analysis of metaphorical language. A particular focus was put on the use and function of religio-culturally charged metaphors towards the societal perceptions of SB, expecting a quite frequent use of them. A general distinction was thereby drawn between references to religious semantics and more descriptive images.

With regard to the comparison of the frequencies of used metaphors in German- and English-language media coverage, our quantitative analysis has some limitations: First, as we did no quantitative significance test of the metaphor use, our findings provide just initial indications of differences between national and international media reporting on SB. Thus, no conclusions can be reached about the significance of the differences in the frequency distribution. Second, there is a large difference in size between the two examined datasets and the English-language corpus can be considered as more disparate than the German one in terms of type of publication, and geographical origin. Hence, we were well aware that the comparability between both datasets is limited.

In order to specify the way forms of metaphorical speech were used, and to identify their respective contexts of meaning, a qualitative content analysis complemented the investigation of media representations of SB. In particular, the analysis focused on implicit qualities, such as emotional language, optimistic or pessimistic connotations, as well as on contextual factors such as scientific events mentioned in the articles. Thus, we examined for example, whether the metaphors were used in an emotional charged or in a distancing, rational-pragmatic manner in terms of the evaluation of (envisioned) scientific developments, which implies a rather sceptical or a rather affirmative assessment of these progresses. The following sections provide an overview of the most relevant findings regarding the analyses of the use of metaphor in national and international public discourse.

## Results

We identified 1036 German-language articles containing the term “Synthetische Biologie”and 10831 English-language articles containing term “Synthetic Biology”. Thus, the media analysis included a total number of 11867 publications dealing with SB, covering the period between 2004 and 2015. The content of the articles ranged from SB as the main topic to articles where SB was mentioned only peripherally. During the research period, the number of national and especially of international articles within this subject area increased substantially, from 5 respectively 33 articles published in 2004 to 74 respectively 2760 articles published in 2015. With regard to the temporal frequency distribution, it revealed that important scientific events—like the successful “creation of a bacterial cell controlled by a chemically synthesized genome” by Craig Venter and his team [32]–considerably intensified the public discourse on SB. As shown in the following section, such pioneering research results, and the related postulations of a generation of new forms of life [33], also increased the use of metaphors in media coverage on SB, which particularly applied to the use of religio-culturally charged expressions.

### 3.1 On the use of religio-cultural metaphors in the media discourse on synthetic biology

The most frequently used religio-culturally charged metaphors and imaginations in both German and English language media coverage were “playing God”, “(man as) creator” and “creating life”–metaphorical constructions that refer to actors and agencies within the field of SB and link perceptions and evaluations of scientists and their research activities to religio-culturally mediated associations of creation [5]. Looking separately at each of these forms of speech, it becomes clear that the metaphor “creating life” (combined with syntactic patterns such as “artificial” or “synthetic” life forms) was most prevalent in the national and international public discourse. In particular, this applied to German-language media coverage, using the phrase in about 14 percent of the examined articles, while the expression appeared in only about 3 percent of the relevant English-language publications (see Table 1). In comparison, the other two metaphors could be found with substantially less frequency in national as well as international media reports (“playing God” in 4 percent respectively in under 1 percent, “(man as) creator” in about 3 percent respectively in about 1 percent of the articles).

**Table 1:** Frequencies of most-used religio-cultural metaphors in the field of SB in German and English language media coverage 2004–2015 (in percent).

Religio-cultural metaphors	German language media coverage (n = 1036)	English language media coverage (n = 10.831)	Combined German and English language media coverage (N = 11867)
Playing God	4.0% (n = 41)	0.7% (n = 79)	1.0% (n = 120)
(Man as) creator	3.3% (n = 34)	1.2% (n = 121)	1.3% (n = 155)
Creating life	13.8% (n = 143)	2.8% (n = 302)	3.7% (n = 445)
Total use of religio-cultural metaphors*	**21.1% (n = 218)**	**4.7% (n = 502)**	**6.0% (n = 720)**

* The total use count includes multiple references to different metaphors per article.

Overall, the quantitative findings mainly show three aspects: The first important result is that the total frequency of religio-cultural metaphors used in public discourse on SB was lower than expected on the basis of other empirical findings [1,19], and the ongoing broad ethical discussion of religiously charged expressions in the field of SB [8,9,21,22]. Taking German and English language media coverage together, such metaphors were used in under 5 percent with regard to each metaphor, and in just 6 percent where all three religio-cultural metaphors together were concerned (see Table 1). As already mentioned above, the only metaphorical expression found quite frequently, which especially played a role in German-language media coverage, was the phrase “creating life”. This leads to the second central result: There was a major discrepancy between the frequency of the use of religio-cultural metaphors in German-language media coverage (21.1 percent) and English-language media coverage (4.7 percent), with a total use of such forms of metaphorical speech that was over four times higher in German-language articles on SB (see Table 1). As already mentioned in the methods section, this difference can be considered as a substantive, practical significance but not as a statistical. Therefore, this finding provides initial indications of differences between national and international media reporting on SB, which should be further statistically investigated with regard to their significance. The third important finding, which applied to both German and English language media coverage, is that remarkable scientific events substantially increased the use of religio-culturally impregnated metaphors in the field of SB. Although, in general, no noticeable percentage increase of the occurrence of religio-cultural metaphors during the research period can be found, in 2010 the use of such forms of speech was considerably more frequent. Combining German- and English-language media reports in 2010, religio-cultural representations were used in 19.4 percent of all articles, which means almost three times as often as the average temporal frequency distribution of such metaphors appearing in the field of SB. As the qualitative analysis of corresponding context factors will show, this can be explained, to some extent, by the public and scientific perception of the research results of the Venter-Institute, and the following visionary claim of a fundamental shift in our technical capabilities blurring the boundaries between what is defined as ‘natural’ and ‘non-natural’.

In order to accomplish a more in-depth examination of the three most frequently observed religio-cultural metaphors in media coverage, the next section will describe the manner in which they were used and their respective contexts of meaning. As the metaphors were used in German and English language articles in a similar manner, the following qualitative findings consistently apply to both examined media reports.

Three modes of use in particular could be identified for each of the religio-culturally charged phrases. Firstly, religio-cultural metaphors served as attempts to find ways to express the present and envisioned technological developments in the field of SB, highlighting uncertainties about the range of possible outcomes as well as the (anticipated) blurring of traditional cultural concepts of ‘life’ and ‘nature’ (a). Secondly, such metaphors were used in a distancing manner in order to relativize the successes and visions of SB and the related claims of a generation of new forms of life (b). Thirdly, the religio-cultural phrases served merely as eye-catching features of newspaper headlines without having any further content-related specifications within the texts (c).

#### ad a)

The metaphors used in the examined media reports often pictured the novel character of SB, its applications and visionary aims, by attributing a religious meaning to the present and anticipated biotechnological developments. In doing so, most of the articles borrowed religio-cultural images to describe the involved scientists (“man as creator”) and especially their research activities (“playing God”, “creating life”). One example of such use is the statement observed with particular frequency in 2010 and 2011 that *“Researchers are creating (new / artificial / synthetic) life (forms)*. ”Accordingly, such metaphors associate perceptions and evaluations of actors and agencies in the field of SB with religio-culturally mediated implications of creation. Although the forms of speech employed refer to ideas from a creation-theological background (in biblical narrations creation being originally defined as an exclusively divine process [8]), commonly the media reports did not reflect their specifically theological content on a metalinguistic level. However, owing to their religious connotations, such metaphors tend to postulate a fundamental shift in the technical capabilities of mankind, transforming the classical distinctions between ‘living’ and ‘non-living’ as well as between ‘natural’ and ‘artificial’ [8,34]. This (possible) rupture of central binary categories can be described as an implicit message in the articles and, again, is hardly ever reflected or discussed with regard to resulting ethical, legal and societal challenges.

Although discussions of the risks and ethical concerns appeared only marginally in media coverage, at least some articles addressed certain anxieties, especially those caused by the success in generating the first synthetic cell in 2010. Within this context, religio-culturally charged metaphors were used to emphasise potential risks and ethical uncertainties, conveying fears that scientists acting as creators are venturing beyond natural boundaries and thereby provoking incalculable consequences for the future. However, rarely have there been explicitly critical statements against SB. On the other hand, however, considerations contrasting possible benefits and risks of applications of SB, as in the following excerpt, were rather common:

“The booming field of synthetic biology holds promises for creating new antibiotics and other drugs. It has also raised concerns scientists are in some way "playing God" by creating living things that could escape from labs into the outside world where they have no natural predators and nothing to check their spread.”

(International New York Times, 8.5.2014).

#### ad b)

Furthermore, the three identified religio-cultural metaphors were used in order to extenuate the current state of scientific applications and successes within the field of SB, as well as the related postulations of a generation of new forms of life. The authors of those media reports argued that, despite the novelty of some proceedings, the scientists still resort to natural structures and functions in their research activities, and then simply modify and combine them in new ways by technical means. Accordingly, many articles stressed that scientists, e.g. the Venter-Institute in 2010, did not create life ex nihilo, but were merely copying already existing forms of natural life:

“Craig Venter has not created life, only mimicked it.”

(The Charlie Rose Show, 21.10.2013).

This attribution also applied to scientific developments expected in the future:

„Doch trotz aller Fortschritte der synthetischen Biologie: Von der tatsächlichen Schaffung von Leben sind Forscher letztlich weit entfernt.“

(Berliner Morgenpost, 18.4.2015).

[Translation: *“But despite all progress made within synthetic biology: Researchers are still far from actually creating life.”*]

As the cited excerpts show, it can be stated that these articles were using religio-cultural metaphors in a distancing manner, so as to highlight that religiously impregnated descriptions, of the involved scientists and their research activities, are not appropriate for representing the actors and agencies within the field of SB. Thus, the German and English language media reports of this category tended to reject allegations of the rupture of boundaries, caused by the successes and visions of SB, that are associated with religio-cultural metaphors such as “playing God”.

#### ad c)

Another very frequent usage of the three religio-cultural metaphors in media coverage serves to draw attention to the respective article. Hence, in several of the examined media reports, such metaphors served merely as teasing, interest provoking features, without having any further content-related specifications or evaluations in the texts. The following headlines of German and English language media reports illustrate this function of religio-cultural metaphors as eye-catchers for attracting public attention:

„Und Craig sah, dass es gut war. Genpionier Craig Venter hat erstmals künstliches Leben geschaffen”

(Financial Times Deutschland, 21.5.2010)

[Translation: *“And Craig saw that it was good. Gene pioneer Craig Venter has created artificial life for the first time.”*]

“Scientist Craig Venter creates life for first time in laboratory sparking debate about 'playing God'”

(The Telegraph, 20.5.2010)

„Die neuen Schöpfer. Biologen konstruieren Lebewesen aus bestehenden Bausteinen“

(Wunderwelt Wissen, 26.4.2013).

[Translation: *“The new creators. Biologists construct living beings from existing bricks.”*]

As the selected quotes suggest, this usage is again closely associated with the context of the generation of a synthetic cell by Venter and his research team. This serves, at least to some extent, to relativize the above mentioned increase of metaphor use in. Beyond expressing the uncertainties evoked by the blurring of traditional cultural concepts of ‘life’ and ‘nature’, the religio-cultural metaphors partly served as mere eye-grabbing features for attracting public attention, without leading to a deeper discussion of ethical issues.

### 3.2 On the use of descriptive metaphors in the media discourse on synthetic biology

As already mentioned, we have drawn a general distinction in the media content analysis between references to religious semantics and images that are more descriptive. In terms of the descriptive metaphors it is important to consider that the categories are not independent, which means that a single metaphorical expression can occur in more than one article (see Table 2). The most frequently used descriptive metaphors in both German and English language media coverage on SB were “building” as well as wordings from the contexts of “engineering”, “IT / Computer” and “art”. Like the religio-culturally charged expressions, these forms of metaphorical speech refer to actors and agencies within the field of SB. By doing so, they link perceptions and evaluations of scientists and their research activities to associations either with the semantic fields of handcrafts, engineering and computer science, or with that of art. It became apparent, with regard to frequency distribution, that the phrase “build” was most prevalent in national (over 55 percent) and international (about 15 percent) discourse, followed by “engineering” (over 45 percent respectively about 12 percent), which was combined with syntactic patterns such as “construct” or “factory”, and “IT / Computer” (about 22 percent respectively about 7 percent), which was combined with syntactic patterns such as “(re-)program” or “circuits” (see Table 2). Metaphorical constructions within the semantic field of “art”, especially combined with the term “design”, were used in almost 26 percent in German-language media coverage and in about 7 percent of the relevant English-language articles. Another metaphor, which appeared with substantially less frequency in both national and international media coverage, but was included in the analysis because it refers to (possible) products of SB (describing novel objects which cannot be found in nature), was the word “Frankenstein”. This term appeared only marginally media in coverage. It was used in only about 8 percent of the German langue media reports and in only about 1 percent of the English language publications. The metaphor of the “living machine”, which also serves as a description for synthetically altered objects, played an even narrower role in public discourse and was observed in under 1 percent of both German and English language articles. Other metaphors we found in German as well as English language media coverage, which could not be considered in greater detail for lack of space, were: “tailor-made” (13.3 percent respectively 5 percent), “tinker” (9.5 percent respectively 1.3 percent), “play” (9.5 percent respectively 1.3 percent), “evolution” (5.9 percent respectively 1.4 percent) and “writing” (5.7 percent respectively 0.6 percent).

**Table 2:** Frequencies of most-used descriptive metaphors in German language and English language media coverage 2004–2015 (in percent).

Descriptive metaphors	German language media coverage (n = 1036)	English language media coverage (n = 10.831)	Combined German and English language media coverage (N = 11867)
Build	57.3% (n = 594)	15.3% (n = 1653)	18.9% (n = 2247)
Engineering (e.g. construct)	47.5% (n = 492)	12.2% (n = 1326)	15.3% (n = 1818)
IT / Computer (e.g. program)	22.2% (n = 230)	7.6% (n = 827)	8.9% (n = 1057)
Art (e.g. design)	25.9% (n = 268)	7.3% (n = 787)	8.9% (n = 1055)
Frankenstein	8.4% (n = 87)	1.3% (n = 142)	1.9% (n = 229)
Total use of des-criptive metaphors*	161.3%* **(n = 1671)**	**43.7% (n = 4735)**	**54.0% (n = 6406)**

* The total use count includes multiple references to different metaphors per article. Values above 100 percent correspond to this multiple use (161.3 percent = 1.6-fold use).

The quantitative findings show three key aspects: The first important result is that descriptive metaphors (except for expressions related to the “Frankenstein” metaphor) have been quite frequently used in public discourse on SB, especially compared to the use of religio-cultural metaphors. With German and English media coverage combined, descriptive metaphors were observed in up to 19 percent of the articles regarding each metaphor, and in 54 percent regarding all five analysed descriptive metaphors (see Table 2). Hence, the total frequency of used descriptive metaphorical constructions was eight times higher than the total frequency of used religio-culturally charged images in the media coverage on SB. The second result is similar to the findings stated above concerning the occurrence of religio-cultural metaphors in public discourse: once again, there was a discrepancy between the frequencies of metaphor use in German and English language media coverage. While German language articles used descriptive phrases about one and a half times per article, such phrases appeared in only about half of the English-language media reports, resulting in a three times higher total use of descriptive forms of metaphorical speech in German-language articles on SB (see Table 2). As we did no statistical test with regard to the significance of the different frequencies of metaphor use in German- and English-language media coverage, this finding should be considered as an initial indication and thus should not be over interpreted. The third important finding is that the temporal frequency distribution of the used descriptive metaphors remained roughly the same. Thus, in contrast to the noticeable increase of the occurrence of religio-culturally charged metaphors caused by remarkable scientific developments, we found a constant number of descriptive metaphors during the research period.

As has already been done in the previous section, the next section will describe the way the aforementioned descriptive metaphors were used to provide a more in-depth understanding of these forms of speech in media coverage on SB. Again, the metaphors were used nearly in the same manner in German and English language media reports. Therefore, the following qualitative findings consistently refer to both.

With regard to the four most frequent descriptive metaphors that are first listed in the table above, two modes of expression can be distinguished in general: On the one hand, (a) descriptive metaphors were used in an objective, rational-pragmatic manner, aiming at describing the novel scientific proceedings and objects. On the other hand, (b) their usage can be classified as emotionally charged, provoking as well as implicitly reflecting a certain anxiety caused by the present and envisioned technological developments in biotechnology and the (possible) blurring of classical concepts of ‘life’ and ‘nature’.

#### ad a)

The descriptive metaphors used in the examined media reports often depicted applications of SB in a simplifying manner, in order to give a broad readership of laypeople a vivid impression of the complex research activities in the field of SB. The metaphorical nature of these expressions tended to be unremarkable. This indicates that they can be characterised as lexicalised metaphors, and thus, as forms of speech which are primarily already part of the common language. Typical examples of this usage of descriptive metaphors are syntactical patterns such as *“build a bacterium”*, *“artificially constructed chromosome”*, *“synthetic circuits*, *which program designer cells”* or *“design new DNA sequences”*. As these illustrations show, the metaphorical phrases refer to specific scopes of application and function as attempts to find concrete representations for research activities within the field of SB and the related technological developments. In this case, then, media reports used descriptive metaphors in an objective and pragmatic manner.

#### ad b)

Furthermore, the usage of descriptive metaphorical language can be viewed as a vehicle for expressing certain feelings of ambiguity, evoked by the successes and visions of emerging biotechnologies and the associated challenges to collective norms and values, such as the rupturing of boundaries between what is defined as ‘natural’ and ‘non-natural’. By linking applications of SB with terms that do not refer to specific scopes of application but to the whole of human existence, German and English language media reports from this category described the technological developments in an emotionally appealing way. Within this context, phrases like *“trying to build life”*, *„genetischer Umbau des Kerns der Natur*”[translation: *“genetic modification of the core of nature”*] or *“changing evolution by designing new software of life”* were common in media coverage. Due to the abstract and generalising link between research activities within the field of SB and novel forms of synthetic life, the articles implicitly addressed changes that affect, challenge and potentially disturb the general understanding of human beings, their self-image and cultural identity. In particular, the expressed uncertainties were based on expected scientific developments in the future, as the following extracts show:

“This is just the beginning stage of being able to program life.”

(The New Yorker, 28.9.2009)

*“The crucial point* […] *is that scientists are now learning how to design life down to the last letter*. *We don’t know enough to be sophisticated as yet but our knowledge is increasing all the time.”*

(The Observer, 20.4.2008)

Even more than these forms of metaphorical speech, the afore-mentioned “Frankenstein” metaphor refers to possible outcomes of SB, attributing frightening effects to the practices and products of emerging biotechnologies within the field. Such fears derive their naming and main contents from the novel by Mary Shelley, published in 1818, that describes a scientist (Victor Frankenstein) who creates a terrifying creature in a scientific experiment—a scenario that has come to be broadly synonymous with technological progress going too far and getting out of control [1,2,35]. Thus, recourse to Frankenstein scenarios in national and international media coverage can be described, firstly, as an appeal to emotions, evoking anxieties by conjuring images of at least partly irresponsible scientists, and the possibly horrifying physical outcomes of their research activities:

„Was die Autorin Mary Shelley Anfang des 19. Jahrhunderts in ihrem Frankenstein-Roman niedergeschrieben hat, haben Venter und sein Team umgesetzt: Sie haben eine lebende Kreatur erschaffen.“

(Südwest-Presse, 28.5.2010)

[Translation: *“Venter and his team have realised what the author Mary Shelley has written in her novel Frankenstein at the beginning of the 19th century: They have created a living creature.”*]

Although, as already stated in the previous section on the use of religio-cultural metaphors, discussions of potential risks and ethical uncertainties made only marginal appearances in both English and German language media coverage, at least some articles directly addressed certain anxieties within the context of Frankenstein scenarios:

*„Offensichtliches Gefahrenpotenzial besteht natürlich darin, dass die ‚Frankenstein’-Winzlinge einmal Reißaus in die freie Natur nehmen könnten*.

(Austria Presse Agentur, Zukunft Wissen, 12.2.2010)

[Translation: *“An obvious risk potential is of course that the ‘Frankenstein’-midgets might escape into the environment.”*]

In the cited extract, the “Frankenstein” metaphor was used to emphasise risks and highlight fears that scientists might lose control of synthetically altered objects and thereby unleash incalculable consequences for the future. In a manner comparable to the usage of religious semantics, an aspect that played a role in such references is the implicit assumption that scientists in some fields of SB are venturing beyond natural boundaries. As a result, uncertainties arise regarding the range of their research outcomes, and there is resultant ambiguity owing to the anticipated blurring of traditional cultural concepts.

Secondly, the Frankenstein metaphor was used in a distancing manner, aimed at extenuating the current state of scientific applications and successes in biotechnology as well as the related postulations of generating new, possibly dangerous and uncontrollable forms of life. Thus, in this context, the frightening scenario of monstrous synthetic products as results of present (and envisioned) research activities was rejected by the media reports. For example, regarding the success of Venter and his team in 2010, the *Daily Mail* noted that what the scientists generated was *“not a Frankenstein’s monster*, *or even a mouse*, *but a bacterium*, *one of the simplest living organisms”*, highlighting the harmlessness of current research results within the field of SB.

## Discussion and conclusion

Metaphors that are prevalent in media coverage influence public views and evaluations of emerging biotechnologies by framing and structuring related discourses and creating powerful images of actors, agencies and scientific outcomes within these fields [2,17,24]. Thus, the provided analysis of metaphors in German and English language media coverage of SB reveals comprehensive insights into societal perception and communication modes of this biotechnology, thereby contributing to an enhanced understanding of public discourses in this highly and controversially debated research field. In what follows, the most important results of the presented study are summarised, compared with findings from other investigations and reflected on with regard to overarching meanings and functions of metaphorical language in treating emerging biotechnologies.

As other studies on metaphors in public discourse on biotechnologies indicate [1,2,19], it can be stated, on a general level, that forms of metaphorical speech have been frequently used in German and English language articles and therefore shape media representations of SB. Basic expressions for describing scientists and research practices in both national and international media coverage were mainly descriptive metaphors originating from the semantic fields of craft (“building”), engineering (“constructing”), IT (“programming”) or art (“designing”). These findings correspond with those of a German-language media analysis, which has already shown that metaphors with mechanical and industrial references are most prevalent in public discourse on SB [19,20]. In comparison, the total frequency of religio-cultural metaphors used in media coverage of SB was substantially lower than the occurrence of those descriptive expressions. The only metaphorical construction found quite frequently, and which especially played a role in German-language media coverage, was the phrase “creating life”. This low usage rate of religio-cultural expressions can be considered a surprising result, especially since other empirical studies [1,19], and particularly the ongoing broad ethical discussion of religiously charged metaphors in the field of SB [8,9,21,22], attach more importance to these forms of speech. Yet, our quantitative analysis shows that leading metaphors in the ethical discourse on new medical or biotechnological procedures in general, such as “Playing God” [22,36], were used with considerably less frequency in media coverage on SB than expected. The same applied to the widely discussed phrases “Frankenstein” and “Living machines” [1,2,21,23], which appeared only marginally in media coverage on SB. The qualitative analysis confirms this rather subordinate role of religio-cultural and other emotionally appealing metaphors, demonstrating that they were at least partially used in a distancing manner. This calls into question whether such phrases are appropriate for representing actors, agencies or synthetically altered objects.

Furthermore, the present data indicate a major discrepancy, between the frequencies of metaphor use in German and English language media coverage on SB, which was found for both religio-cultural and descriptive metaphors. As the analysis shows, it can be assumed that forms of metaphorical speech address the novelty of current and envisioned scientific developments, highlighting their potential to shift social values and cultural concepts of life and nature. According to this function, the more frequent use of metaphors in German language reporting can be explained as resulting from a different perception of the role and meaning of emerging biotechnologies in the German language area [10], where a stronger emphasis is placed on the transformational potential of such scientific progresses. Assuming that religiously or emotionally charged metaphors serve as attempts at finding expressions for novel, border crossing developments in biotechnology, articulating uncertainties and anxieties evoked by scientific progress [2,8,37], it can furthermore be stated that German language articles implicitly transport this ambiguity to a greater extent than English language reports. Interestingly, this can be linked to the findings of large-scale surveys of public perceptions of biotechnology, which indicate that respondents from Germany and Austria express comparably high concerns about SB [10]. In addition, a qualitative and quantitative study amongst Germans reveals that they perceive SB as a complex and difficult issue [11]. Thus, beyond generally supportive public attitudes towards science and technology, especially regarding medical applications [13,37], it can be assumed that there is nonetheless some kind of unease about crossing ethical boarders and blurring traditional cultural concepts within certain developments in biotechnology [38]. In particular, this vague unease is implicitly reflected on a narrative level by the specific manner in which German media coverage uses metaphorical expressions. However, because claims about causal effects of media contents on opinions of audiences (and vice versa) are invalid [2,39], the scope of the present reflections is limited. Media reception studies could serve as means for a more detailed examination of interrelations like these, between media coverage of biotechnologies and public attitudes towards these applications.

A more detailed account of the functions and effects of the metaphors used in public discourse on SB shows that the two successively analysed groups of metaphorical phrases can be differentiated from each other. As just mentioned, the different forms of metaphorical speech express the novelty of (envisioned) scientific developments, emphasising their potential to transform the traditional cultural understanding of life and nature. Depending on the evaluation of this change in values, there are two different functions of metaphors: Whereas religio-culturally and emotionally charged metaphors articulate a perceived rupture of boundaries, evoking certain anxieties, the descriptive metaphors merely articulate an ongoing process of change that does not imply a disturbing transgression of ethical borders.

Accordingly, the religio-cultural metaphors seem to function as indicators of a public ambiguity and unease caused by a number of developments within SB. They can therefore be classified as expressions of a predominantly negative, or at least sceptical evaluation of scientific progress in this field. Inasmuch as these wordings attach religious meaning and value to the practices and products of SB, they convey that scientists have transgressed supposedly fixed limits, deeply rooted in human cultural memory [40], which establish a certain order. This order is considered, or at least felt as something like a primary boundary marker of what humans are allowed to, or should be able to do. Thus, the scientists are suspected of exceeding the limits of mankind, and thereby, of irresponsible behaviour, penetrating into a domain that is believed to be exclusively reserved for the divine sphere [8,22,37]. Apart from matters of security and risk, references to religious semantics thus underline challenges to norms and values, such as the blurring of classical distinctions between ‘living’ and ‘non-living’ respectively ‘natural’ and ‘artificial’. They deliver a warning against disregarding the alleged boundaries of mankind. The results of the media analysis support the hypothesis that religio-culturally charged expressions address the public unease evoked by certain developments in biotechnology. As the empirical data show, the most frequent use of religio-cultural metaphors in both national and international media coverage occurred in 2010 and was caused by the pioneering research results of the Venter-Institute and the related postulations of a generation of new forms of life. This indicates that remarkable scientific developments, with the potential to transform traditional concepts of life, increase the occurrence of religio-cultural metaphors in general.

With regard to the effects of these religio-cultural metaphors, it became apparent, that although the used forms of speech refer to biblical ideas from the background of creation, the media reports commonly did not reflect their specifically theological content on a metalinguistic level. In this way, they transfer a process of creation, originally defined as exclusively divine in biblical narrations, to a profane sphere and, in doing so, confer an inappropriate meaning on research activities in the field of emerging biotechnologies. This quasi-religious misconception inspires fantasies of the omnipotence of human actors, which lead to a mythologizing of the assessments of SB, by falsely alleging that research can conquer a sacred domain [20]. Thus, the religio-cultural metaphors in media reports draw a rather unrealistic picture of emerging biotechnologies and might give rise to unreasonable suspicions or exaggerated fears concerning this research field.

In comparison, the more descriptive images used in media coverage address the novelty of current and envisioned scientific developments by framing them merely as an ongoing process of change, that does indeed have the potential to shift cultural concepts of life and nature, but does not simultaneously imply a disturbing transgression of ethical borders. In doing so, these phrases seem to be more objective, and seem to describe the novel scientific proceedings and objects in a rational, pragmatic manner. Their meaning thus depends on the specific context in which they were used and can be rather affirmative or rather sceptical. Nevertheless, especially due to their mostly mechanical and industrial references, they also generate certain images and fantasies of SB. Firstly, the usage of mechanistic metaphors to denominate simple forms of life engenders exaggerated expectations and assessments of SB. It suggests that researchers are able to genetically construct, program or design a desired trait from scratch and as they wish. In point of fact, the implementation of such prospects, of artificially generating new forms of life, has proven more difficult and complex than engineering metaphors promise [41]. Secondly, the mechanistic and industrial attributions of most of the descriptive metaphors used in media coverage give the impression that the generation of synthetic life results in artefacts that are easy to control [19]. This might lead to “an underestimation of life forms that evolve and interact with nature” [9, 42], suggesting that living organisms can be completely controllable. Such a conception of life tends to reduce its means and potentials, which in turn could have the effect of underestimating the impact of applications of SB for the future.

The provided analysis of metaphors used in national and international media coverage on SB demonstrates the transformational potential of innovative biotechnologies by virtue of their capacity to produce profound changes in their physical and social environments, something which may have substantial implications for shared ways of life [43]. In other words: Biotechnological innovation has an inherent disruptive potential. Disruptive in that sense, that they comprise the potential to shift familiar or even traditional values as well as perceptions on life and nature. The important point is that it is really difficult to predict if such a shift—by which societal actors—will be grasped and interpreted more sceptically or more favourably. Against this background, the results of the current study allow a first glimpse into social processes of perception, social handling of (anticipated) shifts of cultural embedded values and, by extension, possible societal tensions. For the further ethical and sociological debate about SB, it will be important to analyse in more detail the hybridity of values implicitly reflected, on a narrative level, by the metaphorical phrases. Inasmuch as these forms of speech express either feelings of a certain anxiety and unease, or fairly affirmative evaluations of synthetic life, they refer to changing attributions to living matter, and thus, to the general understanding of life and social identity. The challenge of the future is to negotiate a societal consensus regarding the extent and manner of changes in values, as caused by emerging biotechnologies, in order to achieve a broad public acceptance of technological developments. For this purpose, it is required to promote bottom-up activities, ensuring an open and transparent discussion along the lines of a dialogical public engagement in scientific practice.

## References

[1] Holmgreen I-L. Biotech as 'biothreat'? Metaphorical constructions in discourse. Discourse & Society. 2008; 9: 99–119.

[2] Coleman C-L, Ritchie DL. Examining metaphors in biopolitical discourse. Lodz Papers in Pragmatics. 2011; 7: 29–59. doi: 10.2478/v10016-011-0003-8

[3] O’Keefe M, Perrault S, Halpern J, Ikemoto L, Yarborough M, UC North Bioethics Collaboratory for Life & Health Sciences. Editing genes: A case study about how language matters in bioethics. Am J Bioeth. 2015; 15: 3–10. 10.1080/15265161.2015.1103804 26632354

[4] Porcar M, Peretó J, Nature versus design: Synthetic biology or how to build a biological non-machine. Integr Biol. 2016; 8: 451–455. 10.1039/c5ib00239g 26616724

[5] Singh V. Recent advancements in synthetic biology: current status and challenges. Gene. 2014; 535(1):1–11. doi: 10.1016/j.gene.2013.11.025 24269673 10.1016/j.gene.2013.11.025

[6] Braun M, Ried J, Dabrock P. Synthetic Biology and Its Envisioned Significance for Modern Medicine In: Schramme T, Edwards S, editors. Handbook of the Philosophy of Medicine. Berlin: Springer; 2015 pp. 915–926. doi: 10.1007/978-94-017-8706-2_42-1

[7] Klöck G. Synthetic Biology: the next step forward for industrial biotechnology In: Giese B, Pade C, Wigger H, Von Gleich A, editors. Synthetic biology. New York: Springer; 2015 pp. 105–111.

[8] Braun M, Ried J, Dabrock P. From homo faber to homo creator? A theological-ethical expedition into the anthropological depth of synthetic biology. World Views Neth. 2013; 17: 35–46.

[9] Müller O. Synthetic Biology: On epistemological black boxes, human self-assurance, and the hybridity of practices and values In: Boldt J, editor. Synthetic biology: Metaphors, Worldviews, Ethics, and Law. Wiesbaden: Springer VS; 2016 pp. 31–45.

[10] Gaskell G, Stares S, Allansdottir A, Allum N, Castro P, Esmer Y, et al. Europeans and Biotechnology in 2010: Winds of change? A report to the European Commission’s Directorate-General for Research. Luxembourg: European Commission; 2010.

[11] Leopoldina German National Academy of Sciences and Institut für Demoskopie Allensbach. Die Synthetische Biologie in der öffentlichen Meinungsbildung: Überlegungen im Kontext der wissensbasierten Beratung von Politik und Öffentlichkeit. Report from January 2015, Discussion Nr. 3. 2015.

[12] Starkbaum J, Braun M, Dabrock P. The synthetic biology puzzle: A qualitative study on public reflections towards a governance framework. Syst Synth Biol. 2015; 9: 147–157. doi: 10.1007/s11693-015-9182-x 28392848 10.1007/s11693-015-9182-x PMC5383795

[13] Pauwels E. Review of quantitative and qualitative studies on US public perceptions of synthetic biology. Syst Synth Biol. 2009; 3: 37–46. doi: 10.1007/s11693-009-9035-6 . 19816798 10.1007/s11693-009-9035-6 PMC2759427

[14] Pauwels E. Public understanding of synthetic biology. BioScience. 2013; 63: 79–89.

[15] Hart Research Associates. Awareness & impressions of synthetic biology. Report by the Woodrow Wilson International Center for Scholars, Washington; 2013.

[16] Hart Research Associates. Perceptions of synthetic biology and neural engineering. Report by the Woodrow Wilson International Center for Scholars, Washington; 2014.

[17] Nelkin D. Selling Science. New York: W. H. Freeman and Company; 1987 doi: 10.1126/science.236.4804.973-a

[18] Bauer MW, Gaskell G, editors. Biotechnology The Making of a Global Controversy. Cambridge: Cambridge University Press; 2002.

[19] Cserer A, Seiringer A. Pictures of Synthetic Biology: A reflective discussion of the representation of Synthetic Biology (SB) in the German-language media and by SB experts. Syst Synth Biol. 2009; 3: 27–35. doi: 10.1007/s11693-009-9038-3 19816797 10.1007/s11693-009-9038-3 PMC2759430

[20] Gschmeidler B, Seiringer A. “Knight in shining armour” or “Frankenstein’s creation”? The coverage of synthetic biology in German-language media. Public Underst Sci. 2012; 21: 163–73. doi: 10.1177/0963662511403876 22586842 10.1177/0963662511403876

[21] v. d Belt H. Playing God in Frankenstein’s Footsteps: Synthetic Biology and the Meaning of Life. Nanoethics. 2009; 3: 257–268. doi: 10.1007/s11569-009-0079-6 20234875 10.1007/s11569-009-0079-6 PMC2837218

[22] Dabrock P. Playing God? Synthetic biology as a theological and ethical challenge. Syst Synth Biol. 2009; 3: 47–54. doi: 10.1007/s11693-009-9028-5 19816799 10.1007/s11693-009-9028-5 PMC2759421

[23] Matern H, Ried J, Braun M, Dabrock P. Living Machines: On the Genesis and Systematic Implications of a Leading Metaphor of Synthetic Biology In: Boldt J, editor. Synthetic biology. Metaphors, Worldviews, Ethics, and Law. Wiesbaden: Springer VS; 2016 pp. 47–60.

[24] Tammpuu P. Constructing public images of new genetics and gene technology: The media discourse on the Estonian human genome project. Trames: a Journal of the Humanities & Social Sciences. 2004; 8: 192–217.

[25] Lakoff G, Johnson M. Metaphors We Live By. Chicago: University of Chicago Press; 2003.

[26] Austin JL. Zur Theorie der Sprechakte (How to do things with words). Stuttgart: Reclam; 2002.

[27] Berger AA. Media and Communication Research: An Introduction to Qualitative and Quantitative Approaches. Thousand Oaks: Sage; 2000.

[28] Riffe D, Lacy S, Fico F. Analyzing Media Messages: Using Quantitative Content Analysis in Research. London: Routledge; 2014.

[29] Neuendorf KA. The Content Analysis Guidebook, Thousand Oaks: Sage Publications; 2002.

[30] Hijmans E. The logic of qualitative media content analysis: a typology. Communications. 1996; 21: 93–109.

[31] Mayring P. Qualitative content analysis: Theoretical foundation, basic procedures and software solution; 2014. http://nbn-resolving.de/urn:nbn:de:0168-ssoar-395173.

[32] Gibson DG, Glass JI, Lartigue C, Noskov VN, Chuang R-Y, Algire MA, et al Creation of a Bacterial Cell Controlled by a Chemically Synthesized Genome, Science. 2010; 329: 52–56. doi: 10.1126/science.1190719 20488990 10.1126/science.1190719

[33] Venter, JC: The creation of 'Synthia'—Synthetic life. 2010 May 23 [cited 9 March 2017]. In: The Naked Scientists, Science Interview [Internet]. https://www.thenakedscientists.com/articles/interviews/creation-synthia-synthetic-life.

[34] Boldt J, Müller O, Maio G. Synthetische Biologie: Eine ethisch-philosophische Analyse. BBL, Bern; 2009.

[35] Hellsten I. Focus on Metaphors: The case of ‘Frankenfood’ on the web. Journal of Computer-Mediated Communication. 2003; 8 doi: 10.1111/j.1083-6101.2003.tb00218.x

[36] Coady CAJ. Playing God In: Savulescu J, Bostrom N, editors. Human enhancement. Oxford University Press, Oxford; 2009 pp. 155–180.

[37] Ried J, Braun M, Dabrock P. Unbehagen und kulturelles Gedächtnis: Beobachtungen zur gesellschaftlichen Deutungsunsicherheit gegenüber Synthetischer Biologie In: Dabrock P, Bölker M, Braun M, Ried J, editors. Was ist Leben—im Zeitalter seiner technischen Machbarkeit. Freiburg: Verlag Karl Alber; 2011.

[38] European Commission. Responsible Research and Innovation (RRI), Science and Technology. 2013. http://ec.europa.eu/commfrontoffice/publicopinion/archives/ebs/ebs_401_en.pdf

[39] European Commission. Public perceptions of science, research and innovation. 2014. http://ec.europa.eu/commfrontoffice/publicopinion/archives/ebs/ebs_419_en.pdf

[40] Neuman W, Russel M, Just R, Crigler AN. Common Knowledge: News and the Construction of Political Meaning. Chicago: University of Chicago Press; 1992.

[41] Assmann J. Das kulturelle Gedächtnis Schrift, Erinnerung und politische Identität in frühen Hochkulturen, München: Beck; 2007.

[42] Boudry M, Pigliucci M. The mismeasure of machine: Synthetic biology and the trouble with engineering metaphors. Stud Hist Philos Biol Biomed Sci. 2013; 44: 660–668. doi: 10.1016/j.shpsc.2013.05.013 23790452 10.1016/j.shpsc.2013.05.013

[43] McLeod C & Nerlich B Synthetic biology, metaphors and responsibility, Life Sci Soc Policy. 2017; 13:13 https://doi.org/10.1186/s40504-017-0061-y. 28849542 10.1186/s40504-017-0061-y PMC5573707

